# Association between thyroid-stimulating hormone and maternal hemodynamics in hypertensive disorders of pregnancy: an observational study

**DOI:** 10.1186/s12884-019-2556-4

**Published:** 2019-11-01

**Authors:** Yu Liu, Bo Gao, Xin Zeng, Jing Yang, Lei Zhang, Ganwei Xu, Ruizhe Jia, Zhengfeng Xu

**Affiliations:** 1 Department of Obstetrics and Gynecology, The affiliated Obstetrics and Gynecology Hospital with Nanjing Medical University; Nanjing Maternity and Child Health Care Hospital, 123 Tianfeixiang, Mochou Road, Qinhuai District, Nanjing, 210004 China; 2 Department of Clinical Nutrition, Nanjing University Medical School Affiliated Nanjing Drum Tower Hospital, 321 Zhongshan Road, Gulou District, Nanjing, China; 3 Nanjing Maternal and Child Health Institute, 123 Tianfeixiang, Mochou Road, Qinhuai District, Nanjing, China; 4 Department of Science and Education, The affiliated Obstetrics and Gynecology Hospital with Nanjing Medical University; Nanjing Maternity and Child Health Care Hospital, 123 Tianfeixiang, Mochou Road, Qinhuai District, Nanjing, China; 5 Department of Medical Records, The affiliated Obstetrics and Gynecology Hospital with Nanjing Medical University; Nanjing Maternity and Child Health Care Hospital, 123 Tianfeixiang, Mochou Road, Qinhuai District, Nanjing, China; 6 Department of Prenatal Diagnosis, Women’s Hospital of Nanjing Medical University, Nanjing Maternity and Child Health Care Hospital, 123 Tianfeixiang, Mochou Road, Qinhuai District, Nanjing, China

**Keywords:** Hypertensive disorders of pregnancy, Thyroid stimulating hormone, Maternal hemodynamics, Cardiac output, Systemic vascular resistance

## Abstract

**Background:**

Hypertensive disorders of pregnancy (HDP) are characterized by hemodynamic disturbances. Altered thyroid function is a risk factor for poor outcomes of pregnancy. However, the associations between thyroid function biomarkers and maternal hemodynamics during pregnancy in HDP remain unclear.

**Methods:**

From January 2016 to January 2018, pregnant women diagnosed with HDP admitted to the Nanjing Maternity and Child Health Care Hospital were prospectively enrolled in the third trimester. Normally distributed variables were expressed as mean ± standard deviation and skewed variables were expressed as median (25th percentile, 75th percentile). Correlations between thyroid-stimulating hormone (TSH) or free thyroxine (FT4) and maternal hemodynamic parameters were assessed by Pearson’s correlation coefficient and 95% confidence interval (95%CI). Bonferroni’s correction for multiple correlations was performed. Logistic regression models with odd ratio (OR) and 95%CI were applied to confirm the associations.

**Results:**

A total of 163 third-trimester pregnant women with HDP with a mean gestational age of 35.62 ± 2.83 weeks were recruited. The infant birth weight of patients with elevated TSH levels was lower than that of patients with normal TSH levels (2635 ± 867 g vs. 3037 ± 673 g, *p* = 0.002). Reduced cardiac output (CO) was defined as CO < 3.5 L/min. The infant birth weight of patients with reduced CO was lower than that of patients with normal CO (2250 ± 510 g vs. 2890 ± 774 g, *p* = 0.002). TSH levels were significantly and negatively correlated with CO (r = − 0.260, 95%CI: − 0.392- -0.103, *p* < 0.001). FT4 levels were not significantly correlated with any of the maternal hemodynamic parameters (all *p* > 0.05). TSH level (OR = 1.371, 95%CI: 1.086–1.733, *p* = 0.008) was confirmed associated with reduced CO in the logistic regression analysis.

**Conclusions:**

Elevated TSH levels are associated with reduced CO in HDP during the third trimester.

## Background

Hypertensive disorders of pregnancy (HDP) are common complications during pregnancy, with incidence rates of approximately 2–8% [1, 2], and can negatively affect gestational outcomes [1]. At an advanced and more severe stage, preeclampsia (PE), which is characterized by gestational hypertension and nephrotic impairment with proteinuria, is one of the most deleterious gestational hypertensive disorders, with high mortality during pregnancy [3, 4]. Gestational hypertension and PE have similar symptoms and can both lead to intrauterine fetal growth restriction (FGR), still birth, preterm delivery, placental abruption and disseminated intravascular coagulation [3], which seriously threaten the health of both the mother and fetus. However, the exact etiology of HDP, as well as effective prophylaxis and therapeutic targets, remain unclarified [3–6]. The commonly accepted pathogenesis of HDP is the abnormal reconstruction of the spiral artery and placental ischemia [7]. Inflammatory factors secondary to placental ischemia also play a key role in inducing vasoconstriction dysfunction in HDP [5]. Moreover, studies have suggested that obesity and thyroid dysfunction are major risk factors for the development of HDP [8, 9].

Maternal hemodynamic alterations occur in pregnant women with HDP [10, 11]. As part of the process of adaptation to pregnancy, cardiac output (CO) tends to increase, and systemic vascular resistance (SVR) tends to decrease in normotensive women [12]. In contrast, women with HDP fail to undergo this hemodynamic adaptation, with a consequent and significant increase in SVR [10–12]. By conducting a longitudinal study, Ghi et al. postulated that hemodynamic disturbances may occur even in the early stages of pregnancy and can anticipate the development of pregnancy-induced hypertension [13]. Tay et al. discovered that a poor prognosis of HDP, especially in terms of FGR, is significantly associated with declining CO [14]. Peripheral vasoconstriction secondary to maternal systemic inflammation and endothelial cell activation are also considered to be responsible for hypertensive disorders during pregnancy [3]. However, the risk factors for the occurrence of these pathophysiological hemodynamic changes have not been fully investigated.

Thyroid dysfunction frequently occurs during pregnancy [1]. Both hyper- and hypothyroidism can negatively affect the prognosis of pregnancy. Moreover, thyroid dysfunction is considered to be correlated with gestational hypertensive disorders [15, 16]. Wilson et al. discovered that the severity of PE is positively correlated with the level of thyroid-stimulating hormone (TSH). By multivariate analysis, hypothyroidism has been confirmed to be an independent risk factor for severe PE and fetal growth restriction [15–17]. However, the underlying roles of thyroid function in hypertensive disorders during pregnancy are still poorly established. In the general population, thyroid function affects the cardiovascular system [18]. However, the associations between thyroid function and maternal hemodynamics during pregnancy in HDP remained uninvestigated.

## Materials and methods

### Patients

From January 2016 to January 2018, pregnant women with HDP who were admitted to the Nanjing Maternity and Child Health Care Hospital were prospectively enrolled in the third trimester. Data were analyzed retrospectively from our prospectively collected dataset. The diagnostic criterion for HDP was an increase in blood pressure to ≥140/90 mmHg after 20 gestational weeks in previously normotensive women [19]. The elevated blood pressure was confirmed by at least 2 measurements at intervals of at least 4–6 h apart. The exclusion criteria were patients with multiple pregnancies, patients with a history of cardiac disorders or chronic hypertension, patients diagnosed with chronic hypertension in pregnancy, preeclampsia superimposed on chronic hypertension, patients with a history of renal dysfunction, patients with a history of thyroid disorders, patients taking thyroxine substitution medicines before hemodynamic exams, patients taking anti-hypertensive drugs before hemodynamic exams, patients with a history of in-vitro fertilization treatment, patients who consumed alcohol or smoked, patients with other chronic diseases and patients lacking complete medical records. With accordance to the principles of the Declaration of Helsinki, this observational study was approved by the Clinical Research Ethics Committee of the Nanjing Maternity and Child Health Care Hospital. Written consent was obtained from all patients enrolled.

### Maternal hemodynamic monitoring

We applied a noninvasive hemodynamic monitoring technique with electronic impedance cardiography (BioZ CardioDynamics, San Diego, USA) to analyze the circulatory function of HDP patients at enrollment. Electronic impedance cardiography is widely used and enables clinicians to quickly and easily observe the cardiac function and peripheral resistance of patients. The performance of electronic impedance cardiography in pregnancy was validated previously [20], and the protocols of electronic impedance cardiography monitoring have been described [20]. Briefly, patients were asked to remain calm and rest for 30 min before electronic impedance cardiography monitoring and were scanned in the left lateral decubitus position. The measurements of all patients were performed by one experienced sonographer. The recorded hemodynamic parameters included systolic blood pressure, diastolic blood pressure, mean arterial pressure, CO, stroke volume, SVR, ejection time percent, peak velocity of flow, mean pressure gradient and velocity time integral. In compliance with the instructions of the electronic impedance cardiography monitor for pregnancy applied in the present study, low CO was defined as CO < 3.5 L/min, and high SVR was defined as SVR > 1600 dyne×sec/cm^5^.

### Thyroid function measurements

Fasting maternal venous blood samples were obtained on the day of electronic impedance cardiography monitoring. Samples were centrifuged, and supernatants were stored at − 80 °C. TSH was measured using an Elecsys-TSH kit (Roche Diagnostics, Mannheim, Germany, RRID:AB_2756377). FT4 was measured using an FT4 Flex reagent (Siemens, Berlin, Germany, RRID:AB_2801666). Thyroid peroxidase antibody was measured with an E-Anti-TPO kit (Roche Diagnostics, Mannheim, Germany, RRID:AB_10698637) and was graded as positive when greater than 60 IU/ml. The normal reference for TSH in the third trimester was 0.3 to 3.5 mIU/L, as recommended [21].

### Covariates

The demographic information of the patients included age, body mass index (BMI, defined as body weight in kilograms/ square of the body height in meters) before pregnancy, gravidity, parity, gestational age at enrollment and complications of PE. The diagnostic criterion for PE was the presence of HDP with urinary protein above 300 mg/24 h [19]. Laboratory parameters were measured from fasting venous blood samples on the day of electronic impedance cardiography monitoring. The laboratory parameters included the urea nitrogen level, creatinine level, glomerular filtration rate (GFR) and albumin level. The 24-h urine protein levels were also recorded.

### Statistical analysis

Data were analyzed by SPSS version 20.0 (IBM, Chicago, USA, RRID:SCR_002865) and MedCalc Statistical Software version 15.2.2 (MedCalc Software bvba, Ostend, Belgium, RRID:SCR_015044). Normally distributed continuous variables were expressed as the means± standard deviations, skewed data were expressed as medians (25th percentile,75th percentile), and the data were analyzed by independent Student’s t-test and the Mann-Whitney U test, respectively. Categorical variables were expressed as frequencies and proportions, and they were analyzed with the chi-square test (χ^2^ -test). Correlations between thyroid function biomarkers and maternal hemodynamic parameters were assessed with Pearson’s correlation coefficient and 95% confidence interval (95%CI). Bonferroni’s correction for multiple correlations was performed. The maternal hemodynamic parameters that had significant correlations with thyroid function biomarkers per Pearson’s correlation coefficient were stratified for logistic regression, and thyroid function biomarkers were plotted into univariate and multivariate regression models (per the stepwise method) with odd ratio (OR) and 95%CI to confirm the associations. A two-sided *p* < 0.05 was considered statistically significant.

## Results

### Baseline characteristics

The present study consisted of 163 pregnant women with a clear diagnosis of HDP. All patients were enrolled in the third trimester with a mean gestational age of 35.62 ± 2.83 weeks. The mean levels of TSH and FT4 were 3.67 ± 2.21 mIU/L and 11.58 ± 2.15 pmol/L, respectively (Table 1). Regarding the maternal hemodynamics, the mean level of CO was 5.66 ± 1.43 L/min. The proportion of pregnant women with HDP who had CO reductions was 27.6% (Table 2). Patients with reduced CO (Additional file 1: Table S1) had higher rates of low-birth-weight infants (44.4% vs. 24.6%, *p* = 0.013). There were 71 patients with TSH levels above 3.5 mIU/L. The birth weights of the infants of the patients with normal TSH levels and elevated TSH levels were 3037 ± 673 g and 2635 ± 867 g, respectively (*p* = 0.002) and the proportions of low-birth-weight infants were 15.2 and 49.3%, respectively (*p* < 0.001).

**Table 1:** Baseline characteristics

Characteristic	*N* = 163
Age (year)	30.83 ± 5.06
BMI (kg/m^2^)	21.94 ± 4.14
Gravidity (n, %)	
1	104 (63.8)
2	43 (26.4)
≥ 3	16 (9.8)
Parity (n, %)	
0	109 (66.8)
1	50 (30.7)
≥ 2	4 (2.5)
Gestational age at enrollment (weeks)	35.62 ± 2.83
HDP	
Gestational hypertension	86 (52.8)
Preeclampsia	77 (47.2)
Laboratory	
Urea nitrogen (mmol/L)	4.36 ± 1.81
Creatinine (umol/L)	56.17 ± 22.57
GFR (ml/min/1.73m^2^)	132.19 ± 44.77
Albumin (g/L)	32.02 ± 5.65
Urinary protein (mg/24 h)	211 (75, 820)
Thyroid function	
TSH (mIU/L)	3.67 ± 2.21
FT4 (pmol/L)	11.58 ± 2.15
Thyroid peroxidase antibody positive (n, %)	6 (3.7)
Birth weight (g)	2862.07 ± 787.48
Birth weight < 2500 g (n, %)	49 (30.1)
Gestational age at delivery (weeks)	37.49 ± 2.88

BMI: body mass index, HDP: hypertensive disorders of pregnancy, GFR: glomerular filtration rate, TSH: for thyroid stimulating hormone, FT4: for free thyroxine. The values of urinary protein were not normally distributed and were expressed as median (25th percentile, 75th percentile), other parameters were normally distributed and were expressed as mean ± standard deviations

**Table 2:** Maternal hemodynamic monitoring of the study population

Hemodynamic parameters	N = 163
Systolic blood pressure (mmHg)	148.13 ± 18.54
Diastolic blood pressure (mmHg)	94.49 ± 12.27
Mean arterial pressure (mmHg)	112.47 ± 13.73
CO (L/min)	5.66 ± 1.43
Stroke volume (ml/kg)	0.92 ± 0.23
Ejection time percent (%)	47.39 ± 5.96
SVR (dyne×sec/cm^5^)	1632.85 ± 449.04
Peak velocity of flow (m/s)	1.2 (1.00, 1.40)
Mean pressure gradient	2.3 (1.90, 3.20)
Velocity time integral	25 (22, 28)

CO: cardiac output; SVR: systemic vascular resistance. Peak velocity of flow, mean pressure gradient and velocity time integral were skewed distributed and were expressed as median (25th percentile, 75th percentile), other parameters were normally distributed and were expressed as mean ± standard deviations

### Pearson’s correlations between thyroid function biomarkers and maternal hemodynamic parameters

The TSH level was significantly and negatively correlated with CO (r = − 0.260, p < 0.001, 95%CI: − 0.392- -0.103), indicating that cardiac output decreased with increasing TSH levels. The TSH level was significantly and positively correlated with SVR (r = 0.162, 95%CI: 0.009–0.308, *p* = 0.039), indicating that systemic vascular resistance increased with increasing TSH levels. After adjustments for multiple correlations by Bonferroni’s correction, only CO was significantly correlated with TSH. The TSH level was not correlated with other maternal hemodynamic parameters (all *p* > 0.05). The FT4 level was not significantly correlated with any of the maternal hemodynamic parameters (all p > 0.05). The 95%CI for the r values were shown in Table 3.

**Table 3:** Pearson correlation of thyroid function indicators with hemodynamic parameters in overall gestational hypertension patients

N = 163	TSH			FT4		
	r	95%CI (lower, upper)	p	r	95%CI (lower, upper)	p
Systolic blood pressure	0.132	−0.022, 0.280	0.094	−0.056	−0.208, 0.099	0.481
Diastolic blood pressure	0.144	−0.010, 0.291	0.067	0.002	−0.151, 0.156	0.976
Mean arterial pressure	0.117	−0.037, 0.266	0.136	−0.062	−0.213, 0.093	0.435
Peak velocity of flow	0.016	−0.138, 0.170	0.836	−0.097	−0.247, 0.057	0.217
Mean pressure gradient	−0.027	−0.180, 0.128	0.736	−0.108	− 0.258, 0.046	0.169
Velocity time integral	0.018	−0.136, 0.172	0.816	−0.155	−0.302, − 0.002	0.048
CO	−0.260	− 0.392, − 0.103	0.001^***^	−0.072	− 0.228, 0.077	0.361
Stroke volume	0.022	−0.132, 0.175	0.779	−0.111	−0.260, 0.044	0.159
Ejection time percent (%)	−0.095	−0.245, 0.060	0.227	−0.015	− 0.169, 0.139	0.846
SVR	0.162	0.009, 0.308	0.039	0.044	−0.111, 0.196	0.581

Remarks: *** indicated for *p* < 0.001. 95%CI: 95% confidence interval. CO: cardiac output; SVR: systemic vascular resistance. Bonferroni’s correction for multiple correlations: *p* < 0.0025

### Univariate and multivariate logistic regression models for reduced CO

According to the electronic impedance cardiography machine instructions for pregnancy, women with CO < 3.5 L/min were categorized as the CO reduction group. BMI before pregnancy (*p* = 0.046), complications of PE (*p* = 0.044), GFR (*p* = 0.049), albumin level (*p* = 0.033) and TSH level (*p* < 0.001) were significantly different between the CO normal and CO reduction groups (Additional file 1: Table S1). Therefore, they were considered as confounding factors in the adjusted model and were pooled into the univariate regression. Parameters with *p*-values less than 0.05 in the univariate regression were adjusted in the multivariate analysis. After adjustments for confounders, the TSH level (OR = 1.371 95%CI: 1.086–1.733, *p* = 0.008) was confirmed to be significantly associated with CO reduction. (Table 4).

**Table 4:** Association between thyroid function indicators and reduced CO

	Univariate		Multivariate	
	OR (95%CI)	*p*	OR (95%CI)	*p*
BMI	1.140 (1.014–1.281)	0.034^*^	1.161 (1.026–1.314)	0.018^*^
GFR	0.983 (0.968–0.999)	0.032^*^	0.987 (0.970–1.004)	0.141
Albumin	0.934 (0.834–1.046)	0.237		
TSH	1.397 (1.126–1.733)	0.002^**^	1.371 (1.086–1.733)	0.008^**^
Complications of PE	1.092 (0.320–3.721)	0.889		

Remarks: * indicated for p < 0.05; ** indicated for *p* < 0.01. In the study population, 45 pregnant women were with low CO and the remained 118 patients were with normal CO level. Baseline factors with *p* value less than 0.05 in comparison between normal CO and low CO groups (Additional file 1: Table S1) were pooled into univariate logistic regression model. Factors with p-value less than 0.05 in univariate regression were pooled into multivariate logistic regression analysis

This observation demonstrated that elevated TSH levels were associated with reduced cardiac output in pregnant women with HDP in the third trimester.

## Discussion

Previous findings suggested that both overt and subclinical hypothyroidism during pregnancy are associated with a negative gestational prognosis, including the occurrence of gestation induced hypertension and PE, spontaneous abortion, premature delivery, fetal distress, fetal growth restriction and fetal death [15, 16, 22, 23]. A meta-analysis of hypothyroidism in pregnancy showed a 2.4-fold and 1.78-fold increased risk for fetal growth restriction and low birth weight, respectively [22]. However, the underlying mechanisms of this pathogenesis have not been clearly established. Barjaktarovic et al. indicated that thyroid function was associated with placental vascular function and placental hemodynamics during the second and third trimesters [9]. Vsilopoulou et al. suggested that thyroid function hormones play a role in trophoblast cell invasion and placentation [17]. Liu et al. postulated that thyroid function is essential in metabolism and protein synthesis, as well as in tissue differentiation and maturation [22, 24]. Researchers have suggested that even with normal FT4 levels, women with increased TSH levels during pregnancy need higher levels of thyroid hormone to ensure fetal development [25]. Our research demonstrated that patients with elevated TSH levels, which may include those with hypothyroidism during pregnancy, had significantly higher rates of low-birth-weight infants. Moreover, our study also found that the proportion of low-birth-weight infants was also higher in patients with hemodynamic disturbances. Perinatal outcomes were poorer for both women with higher TSH levels and those with reduced CO, which provides clues regarding the potential linkage between TSH and maternal hemodynamic alterations.

Thyroid dysfunction is closely correlated with cardiovascular disorders in the general population [26, 27]. Li et al. conducted a study of 184 patients with nonischemic dilated cardiomyopathy and discovered an association between TSH level and poor cardiac prognosis. Thyroid hormones regulate beta-adrenergic positive chronotropic effects, which may lead to a hyperdynamic state and increase cardiac preload [28]. Hypothyroidism-related systemic vascular resistance and endothelial dysfunction may play a pivotal role in the negative prognosis of cardiovascular diseases [29]. Biondi et al. [30] also suggested that hypothyroidism correlated with increased cardiovascular morbidity and mortality due to its negative effect on cardiac contractility, systemic vascular resistance, and endothelial function. Previous studies have postulated that hypothyroidism, even in the subclinical stage, could change cardiac function by altering sarcoplasmic reticulum calcium-ATPase and the transcriptions of other gene products to affect myocyte contractility and dilation [31]. In addition, the elevation in TSH levels could inhibit the synthesis of the endothelial vasodilators, thereby leading to arterial stiffness [31]. Cardiac output may be reduced by approximately 30–50% in the presence of hypothyroidism, as previously reported [32]. Roef et al. conducted a population-based study and found that alterations in thyroid hormone levels, even within the normal range, contributed to alterations in heart rate and the re-construction of cardiac structure [33]. Systolic blood pressure was also confirmed to be positively associated with TSH [33]. By establishing animal models, Gao et al. demonstrated a positive correlation between the TSH level and contractility index, endothelin. A negative correlation was found with heart rate, systolic blood pressure, the maximal rate of the pressure rise and left ventricular systolic pressure [34]. All of the above studies were based on the general population. The relationship between TSH level and cardiovascular function was also confirmed in new-born infants with congenital hypothyroidism [35]. However, to date, there is no report on the association between TSH level and maternal cardiovascular function during pregnancy. Our research demonstrated that in women with HDP, TSH level is positively correlated with reduced CO, which supports the findings of previous studies.

In the present study, TSH levels were significantly and negatively correlated with CO. These results indicated that cardiac output in HDP declined with the elevation in serum TSH during the third trimester. The physiological adaptation of the cardiovascular system in normal pregnancy was characterized by a significant increase in CO [12, 36], which enables maternal compliance with the increased metabolic demands for fetal growth [37]. The previously reported mean percent increase in CO ranges from 13 to 45% during the first trimester [36]. HDP, as frequent complications during pregnancy, is a leading cause of maternal and neonatal mortality and morbidity [38]. Maternal hemodynamic alteration of patients with HDP was considered as rising in SVR secondary to inflammatory factors induced vasoconstriction dysfunction [38]. Maternal hemodynamic disturbances secondary to inadaptation to normal pregnancy play an essential role in adverse perinatal outcomes and postpartum complications [14]. Although the trends regarding CO alterations in HDP are conflicting between different studies, fetal growth restriction was demonstrated to be associated with inadequate CO [14, 37–39]. One indicated that decreased TSH levels were associated with increased CO and an increased risk of pulmonary hypertension in a general population of females [40]. However, this investigation in the context of pregnancy was not conducted. To our knowledge, this is the first study on the association between TSH and maternal hemodynamics in HDP during pregnancy. The co-existing correlation between elevated TSH and reduced CO may provide new insight into the underlying mechanism of the linkage between hypothyroidism in pregnancy and FGR or low birth weight in HDP patients.

There were several limitations in the present research. First, this study contained a small sample size and was based on a single-center design assessing a Chinese population. Multi-center investigations containing larger sample sizes should be conducted further. Second, the patients in the present study were enrolled during the third trimester, and the conclusions are limited to this trimester. Studies on longitudinal changes in TSH and hemodynamics are warranted in the future. Third, this study lacked a comparison with normotensive pregnancies. It was unclear whether this association existed regardless of whether a woman had a hypertensive pregnancy.

## Conclusions

The research revealed that pregnancies with elevated TSH levels and reduced CO have higher rates of low-birth-weight infants. Elevated TSH levels were associated with reduced CO in HDP women during the third trimester of pregnancy. The co-existing correlation between elevated TSH level and reduced CO may provide new insight into the underlying mechanism of the linkage between hypothyroidism in pregnancy and FGR or low birth weight in HDP patients. Mechanisms of the associations between TSH and reduced CO warrants future investigation.

## Supplementary information

**Additional file 1: Table S1.** Comparison of baseline characteristics between normal CO and reduced CO groups.

## Data Availability

The datasets used during the current study are available from the corresponding author on reasonable request.

## Abbreviations

95%CI: 95% confidence interval
BMI: body mass index
CO: cardiac output
FGR: fetal growth restriction
FT4: free thyroxine
GFR: glomerular filtration rate
HDP: hypertensive disorders of pregnancy
OR: odd ratio
PE: preeclampsia
SD: standard deviation
SVR: systemic vascular resistance
TSH: thyroid stimulating hormone
