# Supplementary material for: Association between thyroid-stimulating hormone and maternal hemodynamics in hypertensive disorders of pregnancy: an observational study
Source: BMC Pregnancy Childbirth. 2019 Nov 1;19:396. doi: 10.1186/s12884-019-2556-4 (PMC6824088; doi:10.1186/s12884-019-2556-4)
Supplement: Supplementary file 1 — Additional file 1: Table S1. Comparison of baseline characteristics between normal CO and reduced CO groups. [file 12884_2019_2556_MOESM1_ESM.docx]

Table S1 Comparison of baseline characteristics between normal CO and reduced CO groups

| Characteristic | Normal CO  N=118 | Reduced CO  N=45 | p |
| --- | --- | --- | --- |
| Age | 30.75±5.08 | 31.77±4.88 | 0.486 |
| BMI | 23.27±0.85 | 23.72±0.78 | 0.046^*^ |
| Gestational age at enrollment | 35.70±2.80 | 35.03±2.71 | 0.297 |
| Gravity (n, %) |  |  | 0.893 |
| 1 | 76 (64.4) | 28 (62.2) |  |
| 2 | 30 (25.4) | 13 (28.9) |  |
| ≥3 | 12 (10.2) | 4 (8.9) |  |
| Parity (n, %) |  |  | 0.943 |
| 0 | 78 (66.1) | 31 (68.9) |  |
| 1 | 37 (31.4) | 13 (28.9) |  |
| ≥2 | 3 (2.5) | 1 (2.2) |  |
| Gestational diabetes | 28 (23.7) | 7 (15.6) | 0.256 |
| HDP (n, %) |  |  | 0.044^*^ |
| Gestational hypertension | 68 (57.6) | 18 (40.0) |  |
| PE | 50 (42.4) | 27 (60.0) |  |
| Laboratory |  |  |  |
| Urea nitrogen (mmol/L) | 4.32±1.83 | 4.73±1.65 | 0.140 |
| Creatinine (umol/L) | 55.39±22.54 | 63.79±22.11 | 0.058 |
| GFR (ml/min/1.73m2) | 134.25±45.16 | 111.82±35.87 | 0.049^*^ |
| Albumin (g/L) | 32.18±5.34 | 30.49±8.20 | 0.033^*^ |
| Urine protein (mg/24h) | 209 (73.5, 811.5) | 262 (109, 889) | 0.462 |
| Thyroid function |  |  |  |
| TSH (mIU/L) | 3.50±2.08 | 5.66±2.70 | <0.001^***^ |
| FT4 (pmol/L) | 11.55±2.12 | 11.94±2.52 | 0.529 |
| Thyroid peroxidase antibody positive (n, %) | 4 (3.4) | 2 (4.4) | 0.754 |
| Birth weight (g) | 2890±774 | 2250±510 | 0.002^*^ |
| Birth weight<2500g (n, %) | 29 (24.6) | 20 (44.4) | 0.013^*^ |

Remarks: * indicated for p<0.05; *** indicated for p<0.001. Age, BMI, gestational age at enrollment, urea nitrogen, creatinine, GFR, albumin, TSH, FT4 levels were normally distributed, and were expressed as mean± standard deviation. They were compared by t-tests. Urine protein was skewed variable and were expressed as median (25^th^ percentile, 75^th^ percentile). They were compared by Mann-Whitney U test. Gravidity, parity, complication of PE, Thyroid peroxidase antibody positive numbers were categorical variables and were expressed as number and proportions. They were compared by Chi square tests.
